# Take-Off Time of the First Generation of the Overwintering Small Brown Planthopper, *Laodelphax striatellus* in the Temperate Zone in East Asia

**DOI:** 10.1371/journal.pone.0120271

**Published:** 2015-03-17

**Authors:** Sachiyo Sanada-Morimura, Akira Otuka, Masaya Matsumura, Tomoki Etoh, Yeqin Zhu, Yijun Zhou, Gufeng Zhang

**Affiliations:** 1 Kyushu Okinawa Agricultural Research Center, Koshi, Kumamoto, Japan; 2 Saga Prefectural Agriculture Research Center, Saga, Japan; 3 Plant Protection Station of Jiangsu Province, Nanjing, Jiangsu Province, People’s Republic of China; 4 Institute of Plant Protection, Jiangsu Academy of Agricultural Sciences, Nanjing, People’s Republic of China; Federal University of Viçosa, BRAZIL

## Abstract

Overseas migration of the small brown planthopper, *Laodelphax striatellus* (Fallén), occurs during the winter wheat harvest season in East Asia. Knowing the take-off time of emigrating *L*. *striatellus* is crucial for predicting such migrations with a simulation technique because winds, carriers of migratory insects, change continuously. Several methods were used in China and Japan from late May to early June 2012 and again in 2013 to identify the precise timing of take-off. These methods included: a tow net trap mounted to a pole at 10 m above the ground, a helicopter-towed net trap, and a canopy trap (which also had video monitoring) set over wheat plants. *Laodelphax striatellus* emigrated from wheat fields mainly in the early evening, before dusk. The insects also emigrated during the daytime but rarely emigrated at dawn, showing a pattern that is unlike the bimodal emigration at dusk and dawn of two other rice planthoppers, the brown planthopper, *Nilaparvata lugens* (Stål), and the white-backed planthopper, *Sogatella furcifera* (Horváth). There was no significant difference in the temporal pattern of take-off behavior between females and males of Japanese *L*. *striatellus* populations.

## Introduction

Long-distance movement is one of the primary reasons for the evolutionary success of insects throughout the world [1]. In some instances, insect species migrate long distances utilizing favorable upper winds, and occasionally even fly over the ocean into another continent, i.e. the painted lady butterfly, *Vanessa cardui* (Linnaeus) [2, 3]. Long-distance migration is problematic especially when some viruliferous insect pests that damage agricultural crops in one area can migrate into new unaffected areas and transmit pathogenic viruses to crops, causing serious damage. Such examples include some aphid species [4]. Other insect pests migrate regularly only in certain regions and seasons. Therefore, it is important to understand the factors controlling the timing and migration routes of migrant insect pests. To understand mechanisms of migration, various methods such as behavior observation, gene analysis, meteorological analysis and radar observation have been employed [5]. For example, extensive investigations on the migration in East Asia of the brown planthopper, *Nilaparvata lugens* (Stål) and the white-backed planthopper, *Sogatella furcifera* (Horváth), major insect pests of rice, have revealed their migration source, migration routes, take-off behaviors and other important aspects of their life histories [6–10].

The small brown planthopper, *Laodelphax striatellus* (Fallén) is another important economic insect pest of rice in East Asia. This insect transmits *Rice stripe virus* and *Rice black-streaked dwarf virus* [11, 12]. The two viruses have recently re-emerged in East Asia, including eastern China and central Japan, since 2000 [13–16]. Most notably, an outbreak of *L*. *striatellus* occurred in Jiangsu Province in China, and viruliferous, first generation progeny of overwintering *L*. *striatellus* occasionally migrated overseas to western Japan and Korea in late May to early June [12]. This short period corresponds to the wheat-harvesting season in the province, when a large number of newly emerged insects of the first generation emigrate from harvesting wheat fields to find new hosts, rice plants, producing mass emigrations that occur only once a year during this season [17]. An outbreak of rice stripe disease, for example, occurred in 2008 along the Japanese western coastal regions after the first generation of an overwintering *L*. *striatellus* population immigrated from overseas into western Japan [18]. In Nagasaki prefecture, one of the most western prefectures, the occurrence of *L*. *striatellus* in that year sharply increased by 126% from the previous year [12, 18].

Prediction of such overseas migrations of *L*. *striatellus* in East Asia provides basic information on the insects’ arrival times and areas to local plant protection stations and farmers, and is one of the most important methods for monitoring possible migrations in advance and for efficiently controlling immigrants and their vector-borne diseases subsequent to mass immigrations. Forecasted arrival timing is used to predict the emigrants’ growth stage or the preferable timing for chemical control. Predicted arrival areas are also used to identify possible areas for investigating the occurrence of the pest and for control. To forecast long-distance migrations of *L*. *striatellus* as accurately as possible, a prediction method has been developed [19]. The method consists of two steps: prediction of the period for insect emigration in eastern China based on the effective accumulated temperature and prediction of insect movement with a migration simulation model during a forecast emigration period [19]. The simulation model needs the initial time of taking off to initiate a calculation. Take-off at dusk and dawn were tentatively used in accordance with the observed behavior of *N*. *lugens* and *S*. *furcifera* [10, 20, 21] since observations of *L*. *striatellus* take-off are limited.

Previously, monitoring of *L*. *striatellus* immigrants into rice paddy fields in Japan was conducted at one- or two-hour intervals in June with yellow water-pan traps placed in the paddy fields. Using this method, catch peaks occurred around 0500–1100 h and 1500–1800 h Japanese Standard Time (JST) [22]. However, this method monitored the insect’s immigration activity into the paddy fields, not its emigration activity, although the catches should be, to some extent, related to the take-off time from wheat and barley fields. In another study, hourly monitoring of *L*. *striatellus* with a light trap was conducted during the wheat harvest season in eastern China and peaks of emigrants were trapped at 2000–2100 h Chinese Standard Time (CST) and small catches were trapped at 0500 h [23, 24]. However, the take-off activity in the daytime and early evening was not investigated by these studies since light traps work only during the night. Furthermore, confirmation was required that *L*. *striatellus* flies at high altitudes in a manner similar to *N*. *lugens*, which ascends to make use of winds at high altitudes for its migration [10].

Insects show various patterns of flight periodicity based on data from about half a million species collected in suction traps on the ground in the UK [25]. Using entomological radar, the number of targets at altitudes in summer typically peaks at dusk and dawn and a daytime high-value period, as well as a nighttime layer on some occasions [26]. Practically, the take-off time directly affects the predicted arrival time and area in a migration simulation, since the winds that carry insects change continuously. For this reason, it is crucial to know when *L*. *striatellus* begins its emigration in order to improve the prediction quality.

This study, therefore, monitored the take-off time of emigration of *L*. *striatellus* in late May to early June in both China and Japan. The monitoring employed several trapping methods at different monitoring heights such as a canopy trap, a suction trap, a pole-mounted net trap, and a balloon-supported or helicopter-towed net trap.

## Materials and Methods

### Monitoring the migration source in China

The survey was conducted both in eastern China and western Japan, where winter wheat and summer rice crops are grown. The first generation of overwintering *L*. *striatellus* populations in Jiangsu Province mostly emigrates from late May to early and middle June [17]. The objective of the survey in China was to characterize the take-off pattern of *L*. *striatellus* at the possible migration source, where the insect’s density was high; however, the number of monitoring locations in the Chinese survey was low due to limited funding. Therefore, the survey in Japan aimed to obtain replicated data on the number of *L*. *striatellus* emigrants, although the insect’s density in Japan was relatively small.

**Fig 1:**
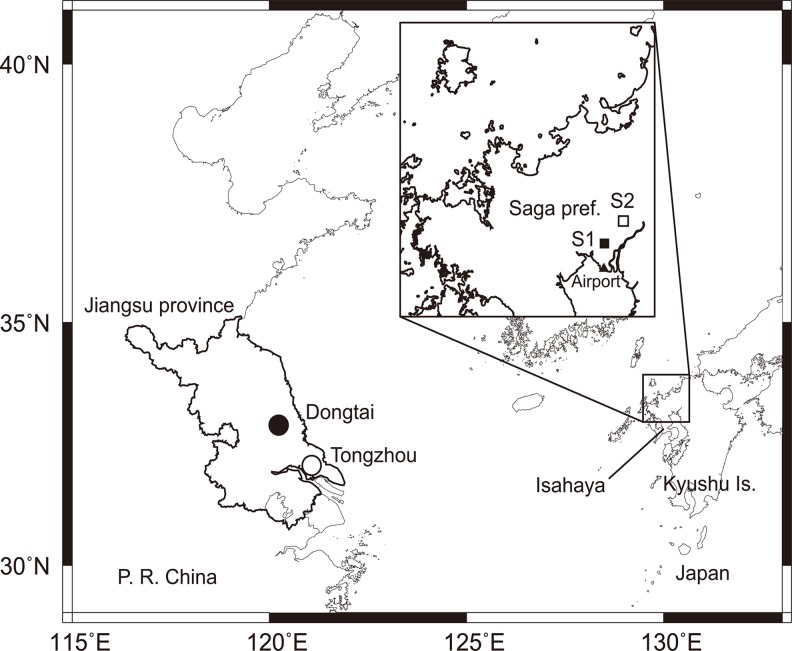
Location map of survey sites.

Two monitoring sites were established in Jiangsu Province, China. Jiangsu is a major occurrence area of *L*. *striatellus* occurrence having had about 70% of the total Chinese *Rice Stripe Virus* occurrence area in 2003 [27], and possibly serving as a source area for the overseas migration into Japan and Korea [12]. One site is located in Tongzhou (32.112°N, 121.077°E, 4 m above sea level (ASL)) in the southern part of the province in 2012, and the other in Dongtai (32.842°N, 120.257°E, 2 m ASL) in the central part in 2013 (Fig. 1). The former site was selected because a net trap was already installed in an experimental field for the Tongzhou Plant Protection Station. The latter site was in an experimental field for the Dongtai Plant Protection Station and was selected because the density of *L*. *striatellus* in wheat fields at 10 locations in the province was checked in the middle of May, 2013 and that in Dongtai was found to be sufficient. The plant protection stations issued permits for land usage. No other special permission was necessary for these surveys because the sites are not located in any protected area and no protected species were sampled in the surveys. A tow net trap was used at the Tongzhou site in 2012 and a canopy trap at the Dongtai site in 2013. Although the tow net trap is one of the standard methods for monitoring migrating insects, tow net traps possibly capture both local emigrants and immigrants from outside of the local area. Therefore, a canopy trap was used in the second year to capture directly only emigrants from wheat fields.

At the Tongzhou site, a tow net trap 1.5 m in depth with a 1-m ring was mounted at the top of a pole 10 m above the ground. The first date of the survey period was determined by referring to the emergence dates of the first generation of the overwintering population. The emergence date was estimated by *L*. *striatellus*’s effective accumulative temperature from the first day of each year with a method described in Zhang et al. (2012) [28]. The stage on the first day was assumed to be the 3rd and 4th instar, and developmental parameters used (the developmental zero, developmental stop temperature and effective accumulative temperature for each stage) were estimates of the Chinese population [28]. Daily air temperature data used were those in Tongzhou obtained from Chinese weather database (http://www.weather.com.cn). The estimated emergence date ranged from May 30 to June 3 and the adult stage ranged from May 30 to June 10, 2012. The first day of the survey began several days before 30 May. The last date of the survey period was determined when large numbers of insects were caught in the net trap. Consequently, insects caught in the trap were collected daily at 0900 h CST from May 25 to June 14, and were collected frequently at 0440, 0700, 0900, 1200, 1500, 1800, 1900, 2000 and 2100 h CST from June 8 to 11. During the frequent monitoring period, insects were not collected when it rained heavily.

**Fig 2:**
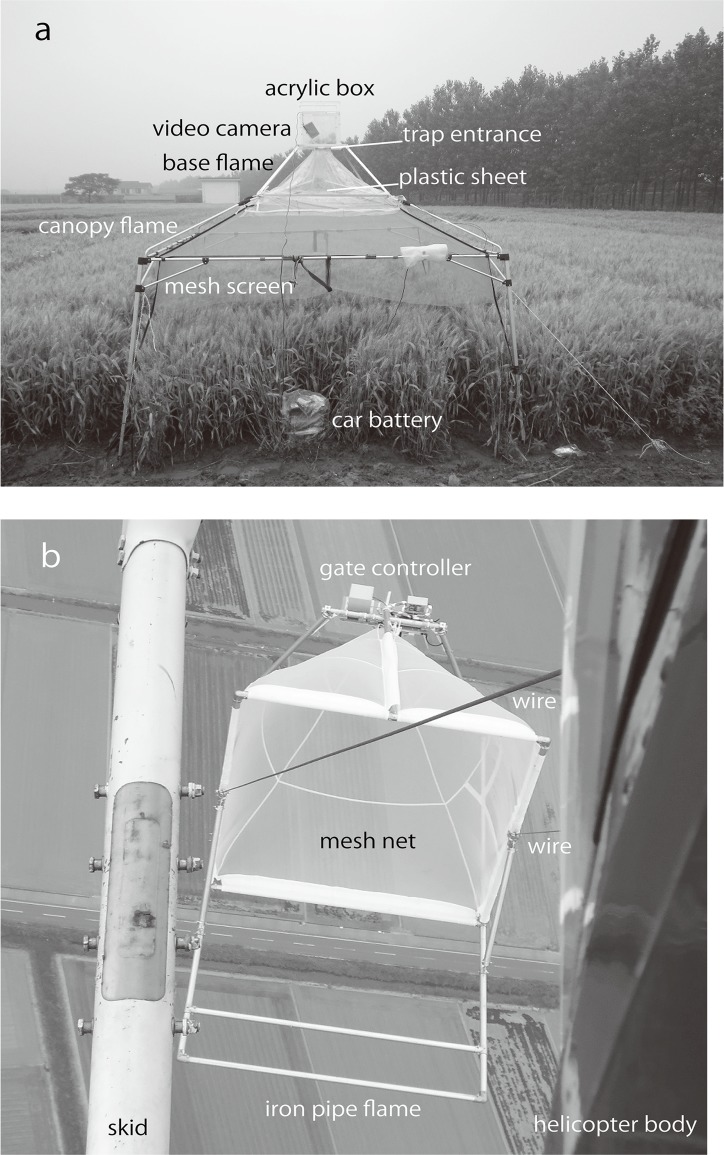
Canopy trap and helicopter-towed net trap. a. Canopy trap over wheat plants. *Laodelphax striatellus* flew directly up into the acrylic box, or insects flew onto the plastic sheet and then walked into the box. b. Helicopter-towed net trap in the air. The photo was taken from the cabin looking below the helicopter. The trap has two gates at the tail of the mesh net. The tail gate was closed when sampling started at about 260 m high, and the front gate closed when the sampling ended. The helicopter maintained its flight speed (65 km/h) and height for 20 min during each sampling.

The investigation period at Dongtai in 2013 was from May 31 to June 6. These dates were determined similarly as described above [28], except that the temperature data at Dongtai were used to calculate the effective accumulative temperatures. The estimate of the emergence date ranged from 3 to 7 June and the adult stage ranged from June 3 to 13, 2013. Two canopy traps (Fig. 2a) to monitor the take-off of *L*. *striatellus* directly from wheat plants were set up over wheat fields at 1700 h CST on May 31, and monitoring was over at 0700 h CST on June 08 (Table 1). Trapped insects were from a natural population at two wheat plots, and no insects were added artificially into the plots from outside. The distance between the two traps was about 50 m. A canopy trap consisted of three major parts: an aluminum canopy frame consisting of a square pyramid (260×260×60 cm) with four 144-cm legs at the corners (Coleman Japan Co., Ltd., Easy Canopy Ultra Light / 260), a mesh screen (Coleman Japan Co., Ltd., Mesh Screen Hanger for Easy Canopy Ultra Light / 260) suspended from the canopy frame, and an transparent acrylic box (30×30×30 cm) with a square hole (65×65 mm) at the bottom, placed on an aluminum base frame over the top of the canopy frame (Fig. 2a). The trap entrance (the square hole, about 220 cm above the ground) and a square aperture (120×120 cm) of the mesh screen at its top were connected with a transparent plastic sheet. The mesh screen was cut horizontally at about 25 cm above the top of the wheat plants to prevent insects from walking in directly from the wheat plants to the mesh. During the monitoring period, the number of *L*. *striatellus* caught in the canopy trap was counted at 0700 and 1900 h CST daily by two of the authors. It took about 35 min to collect insects, change the memory card and video recording mode (see below), and to put the acrylic box back on top of the canopy trap. Therefore, insects were not collected during the periods of 0700–0735 and 1900–1935 h CST (Table 1). The insects collected were put in a refrigerator at -20°C for some time and counted to determine their flight activities in day and night times.

**Table 1:** Canopy trap settings at Dongtai, Jiangsu province, China.

Date	Sample collection time (h)	Video recording mode			Remark
		Period	CT1	CT2	
May 31	-		-	-	Installation at 1700
Jun 1	0700		-	-	
	1900		-	-	
Jun 2	0700	0735–1900	-	Visible	Recording started at 0700
	1900	1935–0700	Infrared	Infrared	
Jun 3	0700	0735–1900	Visible	Visible	
	1900	1935–0700	Infrared	Visible	
Jun 4	0700	0735–1900	Visible	Visible	
	1900	1935–0700	Infrared	Visible	
Jun 5	0700	0735–1900	Visible	Visible	
	1900	1935–0700	Infrared	Visible	
Jun 6	0700	0735–1900	Visible	Visible	
	1900	1935–0700	Infrared	Visible	
Jun 7	0700	0735–1900	Visible	Visible	
	1900	1935–0700	Infrared	Visible	
Jun 8	0700		-	-	Monitoring ended at 0700

In addition, a digital video camera (Sony Corporation, HDR-CX720V) was placed in the box to record the time of each insect’s entry through the hole. The video camera on canopy trap 1 (CT1) used the visible light mode for recording during the day from 0700 to 1900 h CST, and the infrared light mode during the night from 1900 to 0700 h CST (Table 1). The video camera on canopy trap 2 (CT2) mostly used visible light to record insects at dawn (Table 1), because no insects were recorded in the infrared after 0500 h CST due to the saturation of infrared images by the brightness of morning sunlight. No insects were recorded in the visible light mode during the night due to insufficient lighting. The number of insects collected from the canopy traps and seen on the video were not compared and were dealt with separately, because some insects that flew directly into the canopy trap could not be recognized in the video due to their fast movement.

### Monitoring in Japan

The Japanese survey was conducted to monitor *L*. *striatellus* emigrants at different altitudes in two locations for two years. Two monitoring sites were established in Saga Prefecture, western Japan (Fig. 1). Site 1 (S1) is located at 33.218°N, 130.311°E, 2m ASL at the Saga Prefectural Agriculture Research Center who issued the permit of land usage. Site 2 (S2) was located 10.9 km northeast of S1 (33.294°N, 130.385°E, 1 m ASL) and is a private property whose owner issued the permit for land usage. No other special permission was necessary for these surveys because the sites are not located in any protected area and no protected species were sampled in the surveys. Both sites were surrounded by wheat and barley fields that were ready for harvest. Surveys to monitor *L*. *striatellus* emigration were conducted from late May to early June in 2012 and 2013 (Table 2). The first day of the survey periods for both years was similarly determined by *L*. *striatellus*’s effective accumulative temperatures as described above, except using developmental parameters of the Japanese population [19]. Air temperature data used were those from Saga Weather Station about 3.2 km north of S1. Estimated emergence dates in 2012 and 2013 were 27 May and 28 May, respectively, and the survey period for each year started a few days before the corresponding estimate (Table 2).

**Table 2:** Monitoring methods and parameters.

Location	Monitoring method	Survey period^a^		Operation hours (JST) / number of flight	Sampling intervals /sampling duration per flight
		May 26-June 5, 2012	May 24-June 8, 2013		
Site 1 (S1)	Net trap at 10 m	P	P	24 h	1,2 h
	Net trap at 5m		P	24 h	1,2 h
	Balloon-towed net trap at 20 m	P	P	0430–2100	1,2 h
	Suction trap at 1.7 m	P^b^	P	24 h	1,2 h
	Light trap at 1.5 m	P	P	1800–0600	Daily
Site 2 (S2)	Balloon-towed net trap at 10 m	P	P	0430–2100	1,2 h
	Suction trap at 1.7 m	P^b^	P	24 h	1,2 h
	Light trap at 1.5 m		P	1800–0600	Daily
Airport	Helicopter-towed net trap at 260 m		P^c^	6 flights	20 min

^a^ P: Each monitoring was performed.

^b^ Operation in 2012 was limited from 0430 to 2100 h.

^c^ Suvey date: May 31, 2013.

Several types of traps were used to capture insects at different altitudes (Table 2). At S1 in 2012 and both sites in 2013, a light trap with a 60 watt electric bulb at 1.5 m above the ground (AGL) and a funnel beneath the bulb (Ikeda Scientific Co., Ltd., MT-7-N) were used. The trap was operated from 1800 to 0600 h JST to collect insects during the night.

The suction trap was a Johnson-Taylor type [29] painted yellow; a net 80 cm in depth with a 60-cm circular aperture was mounted at 1.7 m above the ground to a vertical circular cylinder 1 m long and 70 cm in diameter, under which a 400-watt fan 60 cm in diameter (Mitsubishi Electric Co., Ltd., EG-60ETB-PR) was installed at 70 cm above the ground to move air into the cylinder (9210 m^3^/h). Suction traps were operated during 0430–2100 h JST in 2012, and for 24 h in 2013 (Table 2).

The 10-m net trap was the same type as that used in Tongzhou, China and was used only at S1 (Table 2). In 2013, an additional net trap of the same size was mounted at 5 m above the ground on the same pole as the 10-m net trap, in order to catch insects at a lower height. The net traps were operated for 24 h.

The balloon-suspended net trap consisted of a helium-filled captive balloon 2.3 m in diameter and a tow net trap 80 cm in depth with a 60-cm ring attached to a supporting rope under the balloon. The height of the balloon-suspended net trap at S1 and S2 was 20 and 10 m AGL, respectively. The balloons were lowered for safety to 2 m AGL both during the night (2100–0430 h JST) and when the wind was strong or it rained.

Insects in the 10-m or 5-m net traps, suction traps and balloon-suspended net traps mentioned above were collected at 0430, 0600, 0800, 1000, 1200, 1400, 1600, 1800, 1900, 2000, and 2100 h JST, and the number of trapped *L*. *striatellus* was counted. For the traps at S1 and S2, data on days when there were missing data due to strong wind, heavy rain, and electric power interruptions were excluded.

Statistical analysis of the Japanese trap data consisted of three steps. First, to avoid sparse data from the catch number in each time category, catch number data of female and male *L*. *striatellus* were re-categorized into 3 time categories, i.e., time category from 0600 to 1200 h JST (Morning), at 1400 and 1600 h JST (Afternoon), and at 1800 and 2000 h JST (Evening). Then, the overall difference between the catch pattern of females and males was analyzed by logistic regression and likelihood ratio tests with times (Morning, Afternoon, Evening) nested by 5 different traps (the 10-m net trap at S1 in 2012, the suction trap at S2 in 2012, the 10-m net trap at S1 in 2013, the 5-m net trap at S1 in 2013, and the suction trap at S1 in 2013). These traps were selected because their catch numbers were large, and daily catch numbers ≧10 were used for each trap. In the second step of the analysis, when it was evident that there was not any significant difference between females and males, the numbers of female and male were summed for a simple insect catch number within each original time category (0600, 0800, 1000, 1200, 1400, 1600, 1800, and 2000 h JST) on a daily basis. Then, proportional data of *L*. *striatellus* (sum of the number of females and males in each time category/ daily total number of females and males) were arcsine transformed, and analyzed to determine overall differences among the eight time categories (0600, 0800, 1000, 1200, 1400, 1600, 1800, and 2000 h JST) and five different traps by a General Linear Model. In the third step, when there was a significant difference among the time categories and/or traps in the previous step, differences in the arcsine-transformed proportion data between a pairwise combination of the time categories and/or traps were determined by the Tukey-Kramer HSD test. These statistical analyses were conducted with JMP 8 software (SAS Institute Inc., Cary, NC, USA).

The helicopter-towed net trap consisting of a square pyramid of mesh fabric was used to catch insects at an altitude as described by Beerwinkle et al. (1990) [30] (Fig. 2b). The frame of the net trap was made of steel pipes (48.6 mm in diameter, JIS G3444 STK500) and zinc alloy joints. Steel wires (9 mm in diameter × 10 m long) connected the net trap and a 1-m balance (Supertool Co., Ltd., LSBN11) that was attached to a load hook on the helicopter (Eurocopter, AS350B). The size of the square aperture of the plastic mesh net was 207×207 cm and the depth was 226 cm. A small mesh bag to catch insects was attached with a hook and loop fastener to the square mesh tube (8×8×20 cm) at the tail of the net. The mesh bag and the mesh tube were closed by two clipping gates on each slider unit (Oriental Motor Co., Ltd., ELSM2XE020K), a tail gate and a front gate, being controlled by an operator in the helicopter’s cabin with a tablet computer (Google Inc. Nexus 7) through a wireless LAN. During ascent for a sampling flight, all the gates were open. The tail gate was closed when the helicopter reached a level of about 270 m above the ground and sampling at about 260 m started. During the sampling, the helicopter maintained its height and flight speed (65 km/h) and followed an elliptical flight-path (3 km EW × 1.7 km NS) for 20 min. The center of the flight-path was located over wheat fields about 3 km north of Saga Airport (33.150°N, 130.302°E: 7.7 km south of S1) (Fig. 1). The front gate was closed when the sampling ended. Six flights with starting times of 0718, 0832, 1031, 1530, 1647 and 1826 h JST on May 31, a sunny day, were conducted. There was no repetition on another day due to limited availability of the helicopter.

The insects collected by the net, suction, balloon-supported and helicopter-towed net traps were frozen in a refrigerator at -20°C and counted within a few days after the collection in a laboratory at the Saga Prefectural Agriculture Research Center.

*Nilaparvata lugens* takes off at an illuminance of approximately 100 lux [19], whereas there were no such reports for *L*. *striatellus*. Hence, the illuminance (lux) at S2 at dusk and dawn was measured in 2012 by an illuminance meter (Sanwa Electric Instrument Co., Ltd., LX2) with the photo sensor pointed upward.

Additionally, in order to determine whether any overseas immigrants arrived in the Japanese survey area during the two-year survey period, simulated migration from Jiangsu Province to western Japan was examined within 24 h after taking off. In other words, this analysis showed whether winds favorable for the overseas migration from China to western Japan occurred. The migration simulation was calculated using the prediction method reported in Otuka et al. (2012) [19], and a migration duration of 24 h was a criterion in the method. In addition, the number of insects trapped in an operational 10-m net trap at the Nagasaki Plant Protection Station, Isahaya City, Nagasaki Prefecture (32.837°N and 130.021°E, 2 m ASL) (Fig. 1), located approximately 50 km southwest of the S1 site, was used to determine if any overseas immigrants were trapped there. The net trap was located in the western part of Kyushu Iskand, which is a sensitive place to catch the insect’s overseas immigration. This survey was conducted by the Nagasaki Plant Protection Station in a regular monitoring program. No special permission was needed for this survey because the site is not located in any protected area and no protected species were sampled in the survey

## Results

### Chinese survey

**Fig 3:**
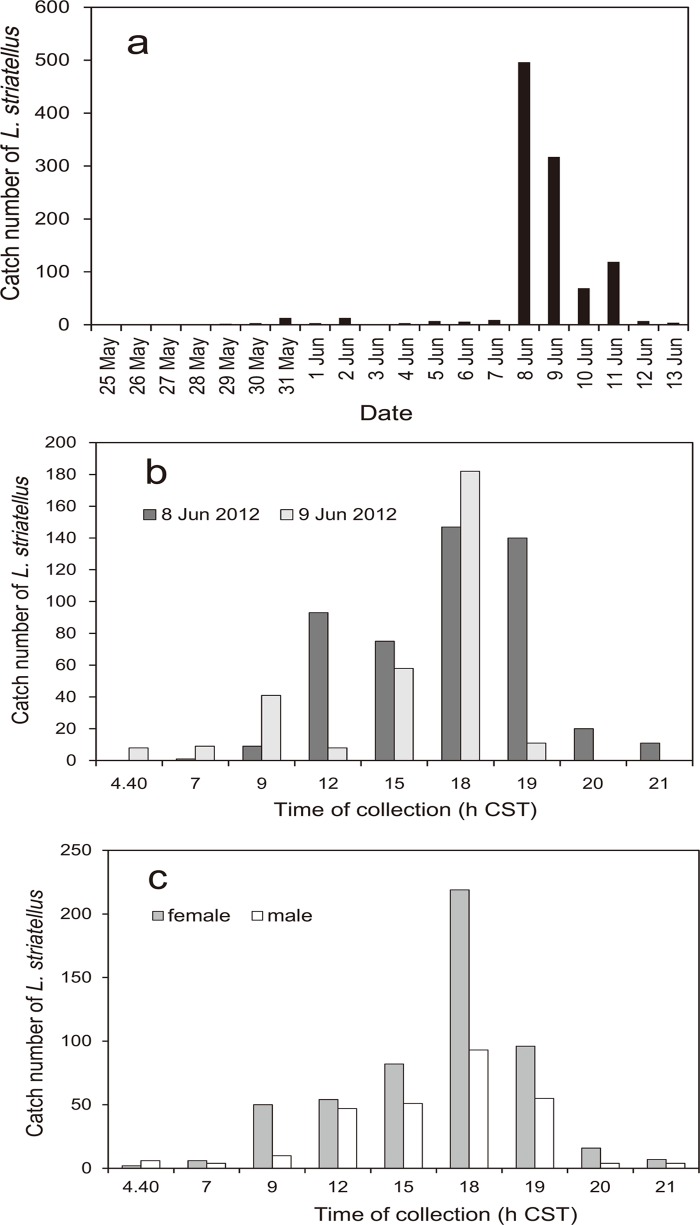
The number of *L*. *striatellus* trapped in the net trap at Tongzhou, Jiangsu Province, China. a. The daily number of insects trapped. b. The number of insects trapped at three-hour intervals on June 8 and 9, 2012. **c**. The sex of insects trapped on June 8 and 9, 2012 as shown in a.

The daily number of *L*. *striatellus* caught in the net trap at Tongzhou in 2012 was low, not more than 13 insects, until June 7. The catch number peaked at 496 on June 8, and then decreased to 317 insects on June 9, 69 on June 10, 119 on June 11, and 7 on June 12. Since insects were not collected at all observation times on June 10 and 11 due to heavy rains, complete hourly catch data were obtained only on June 8 and 9 (Fig. 3a). The number of trapped insects peaked at 1800 h CST (Fig. 3b), and then decreased quickly to a small number at 2000 h CST. Although more female insects were trapped than males (Fig. 3c), the temporal patterns of both sexes were similar, and peaked at 1800 h CST. Sunset at Tongzhou on June 8, 2012 occurred at 1901 h CST, and dusk, when the illuminance is 100 lux, should be about 10 min after this time. There were also some catches during the daytime, from 0900 to 1500 h CST, but catches during the night (from 2100 to 0440 h CST) and dawn (from 0440 to 0700 h CST) were relatively small. Sunrise at Tongzhou on June 8, 2012 occurred at 0449 h CST.

**Fig 4:**
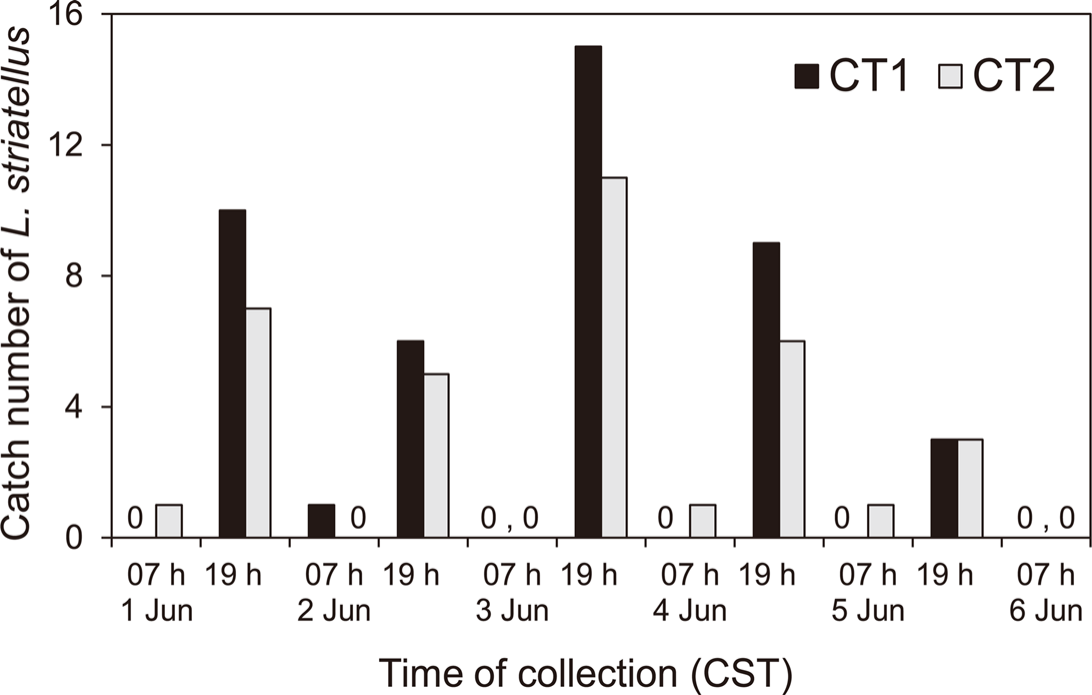
The number of *L*. *striatellus* trapped in the canopy traps at Dongtai, Jiangsu Province, China. The number of insects trapped at twelve-hour intervals from June 1 to June 6, 2012.

**Fig 5:**
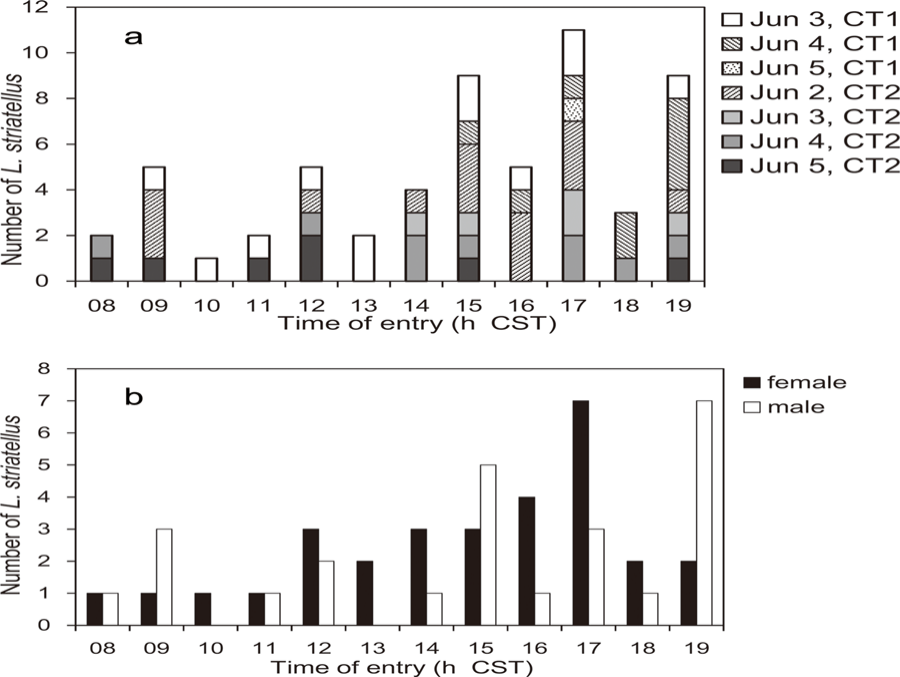
The number of *L*. *striatellus* entering canopy traps at hourly intervals. a. The total number of insects entering canopy traps at two sites, CT1 and CT2. Insects were counted by viewing recorded video images of the trap’s entrance hole. b. The sex of trapped insects as shown in a. Insects of undetermined sex were excluded. For example, 1700 h CST corresponds to the number of insects entering the trap from 1600 to 1700 h CST.

In 2013, few insects were trapped in canopy traps during the night, at dawn or in the early morning (1935–0700 h CST); however, more insects were trapped during the day and evening than at other times (Fig. 4). The total number of insects trapped in the two canopy traps was 79 (female:40, male:39). During the day and evening, 0735–1900 h CST, the number of *L*. *striatellus* that entered the canopy trap changed hourly, but increased gradually towards the evening and peaked at 1700 h (Fig. 5a). In canopy traps, the catch pattern for females peaked at 1700 h, but that for males peaked at 1900 h (Fig. 5b).

### Japanese survey

In the field survey, no *L*. *striatellus* were captured at 0430 h JST in the net trap at S1 in 2012. No *L*. *striatellus* were captured at 0430 h in the 10-m and 5-m net traps at S1 in 2013. Two and one *L*. *striatellus* were caught at 0430 h in the suction trap in 2013 at S1 and S2, respectively. No *L*. *striatellus* were caught by the light traps, except for two specimens caught at S1 in 2013. These results indicate that most *L*. *striatellus* in these two seasons did not fly during the night between 2100 to 0430 h JST.

**Fig 6:**
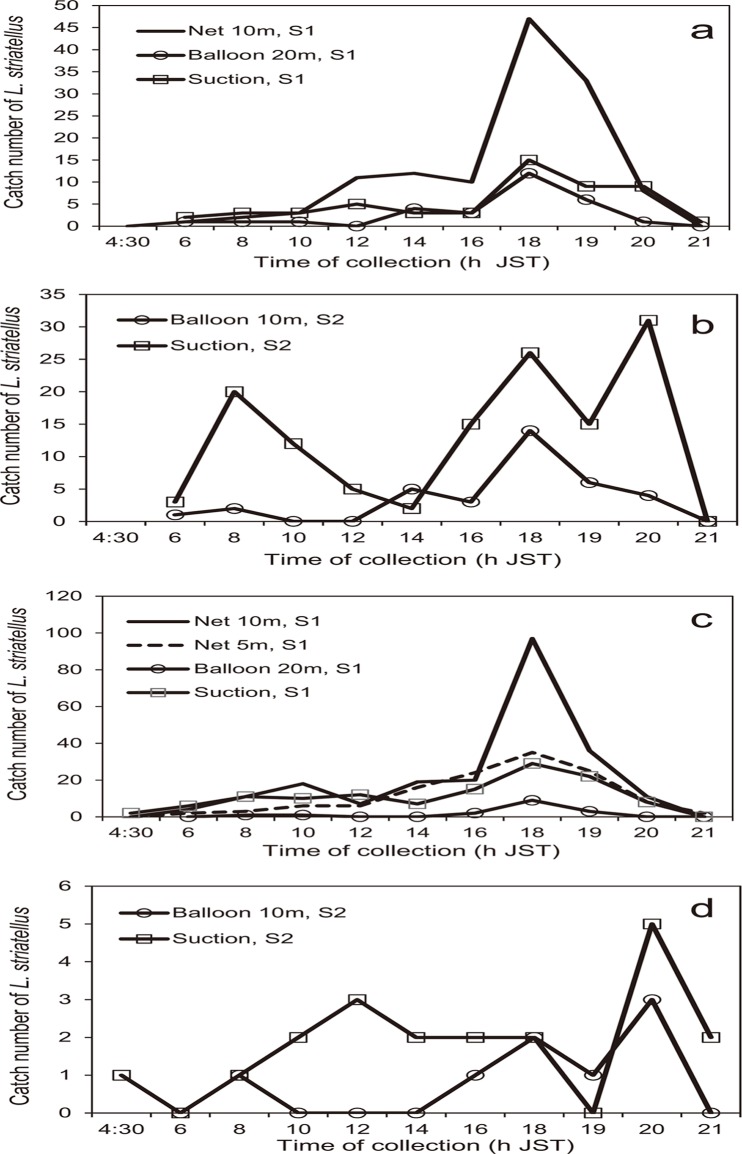
One- or two-hour profiles of numbers of *L*. *striatellus* caught in several types of traps located in Saga Prefecture, Japan. a. S1 site in 2012, b. S2 site in 2012, c. S1 site in 2013, and d. S2 site in 2013. The survey parameters of the traps are shown in Table 2. The number of the insects caught in the light traps is not shown due to their small numbers.

**Fig 7:**
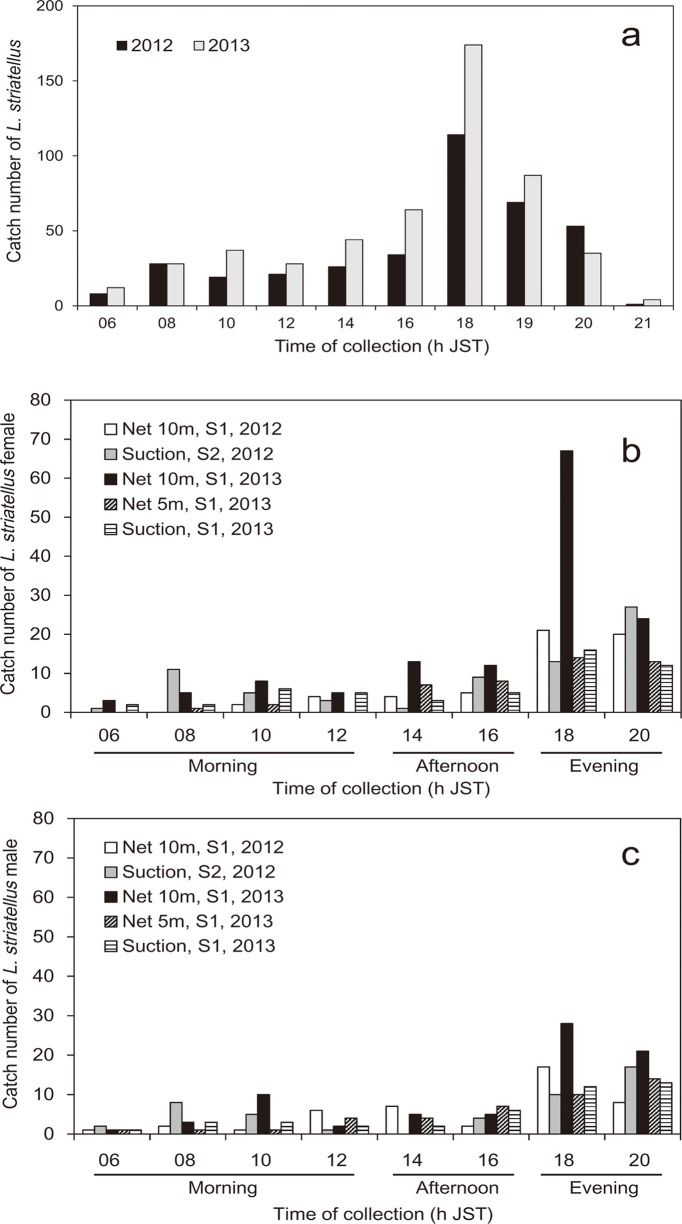
Summary of the Japanese survey. a. The total number of *L*. *striatellus* caught in traps located at both S1 and S2 for each survey year. b. The catch number of *L*. *striatellus* female caught in the 10-m net trap at S1, the suction trap at S2 in 2012, and in the 10- and 5-m net traps and the suction trap at S1 in 2013. These traps were selected because their total numbers were large (> 100) and only days when the daily catch number was more than 10 were used to make the graph. c. The catch number of *L*. *striatellus male* caught in the five traps listed above.

**Fig 8:**
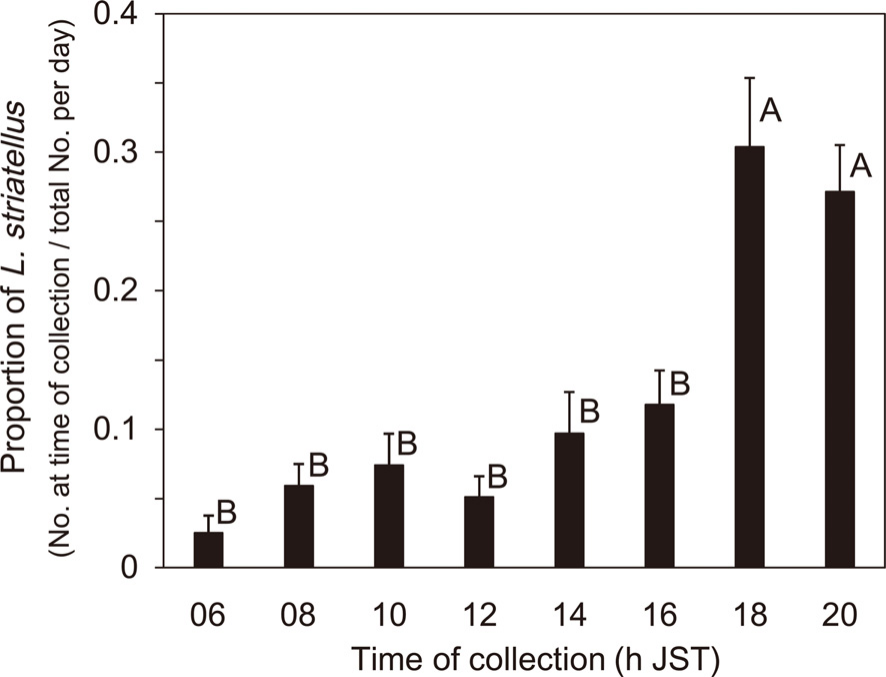
Proportion of *L*. *striatellus* at bi-hourly intervals. Solid bars indicate mean value of the proportion of *L*. *striatellus* (sum of the number of females and males trapped in each time category/daily total number of females and males) for the five traps (the 10-m net trap at S1, the suction trap at S2 in 2012, and in the 10- and 5-m net traps and the suction trap at S1 in 2013) and the two years (2012 and 2013). Error bars indicate the S.E. Different letters indicate significant differences (Tukey-Kramer HSD test, *P*<0.05).

The maximum catch of *L*. *striatellus* for each type of trap, except those of the suction trap at S2 in 2012 and the traps at S2 in 2013, occurred at 1800 h, although some insects were trapped during the day (Fig. 6). Two traps with exceptional patterns at S2 had catch peaks at 2000 h (Fig. 6b and d); however, the catch numbers at S2 in 2013 were very small. The total number of *L*. *striatellus* caught in all the traps at the two sites for each year from 0600 to 2100 h JST had a peak at 1800 h (Fig. 7a). The sum of the catches from 1800 to 2000 h for the two years was equal to 60% of the total catch. There were some peaks during the day (0800–1600 h) (37% of the total), and the number of insects trapped after 1200 h increased toward 1800 h. The number of insects trapped at 0600 h that included catches at dawn was very small. There was no significant difference between females and males among the three time categories (df = 2, χ^2^ = 3.8, ns) and the five traps (df = 12, χ^2^ = 12.7, ns) (logistic regression and likelihood ratio tests) (Fig. 7b, c). The proportional data of *L*. *striatellus* (sum of the number of female and male insects in each time category/daily total number of females and males) converted with the arcsine transformation showed significant differences among the eight time categories (df = 7, χ^2^ = 95.7, *P*<0.001), but not among the traps (df = 32, χ^2^ = 16.9, ns) (GLM test). The maximum and second catch peaks of *L*. *striatellus* at 1800 and 2000 h JST, respectively, were significantly different from other time categories (Tukey-Kramer HSD test, *P*<0.05) (Fig. 8).

Illuminance measurements of 100 lux at dawn occurred at 0501 h JST on two sunny days (May 28 and 29) and one cloudy day (June 3) at the S2 site in 2012. The sunrise on May 28, 2012, for example, happened at 0511 h JST. Illuminance of 100 lux at dusk occurred at 1929 h and 1932 h JST on May 27 and June 3, 2012, respectively. The sunset time on May 27, 2012 was 1920 h.

With the helicopter-towed net trap, one adult was caught in the sampling that started at 0832 h JST on May 31, 2013 and five adults were trapped at the 1826 h JST sampling. No *L*. *striatellus* were caught in the other sampling flights.

**Fig 9:**
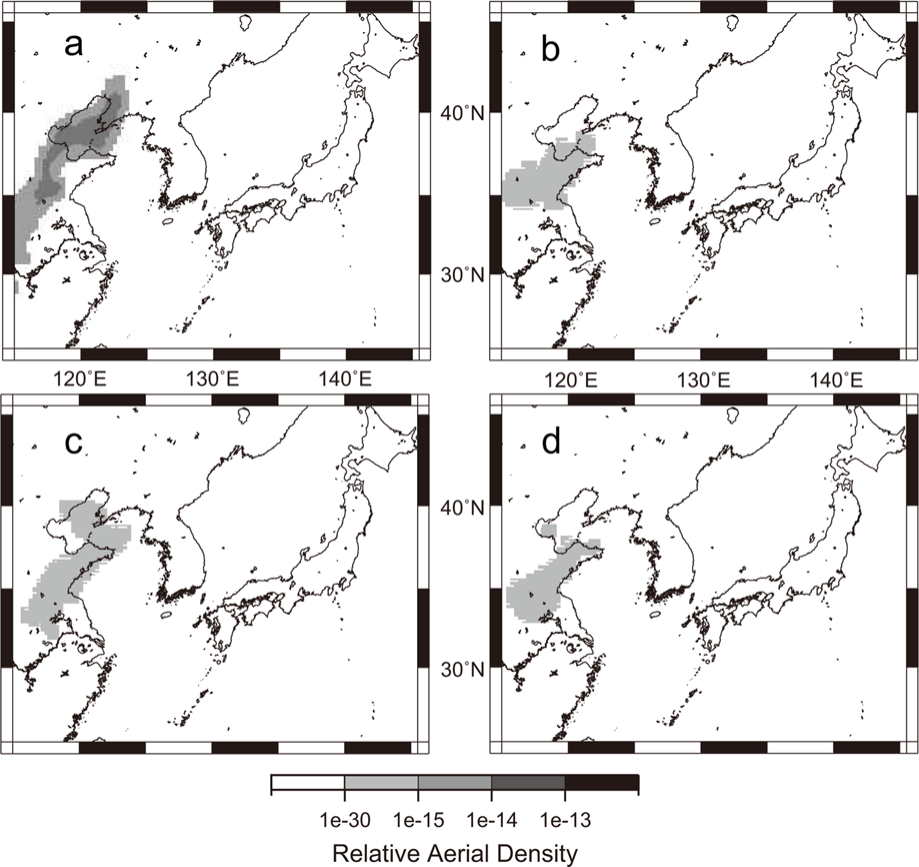
Examples of *L*. *striatellus*’s migration simulation for selected dates during the survey periods in 2012 and 2013. a. May 30, 2012, b. May 24, 2013, c. June 3, 2013 and d. June 4, 2013. These dates are when a small number of *L*. *striatellus* was captured by the net trap at the Isahaya Plant Protection Station, western Japan. In the simulation, *L*. *striatellus* took off in Jiangsu Province on the previous day at 1800 h CST, and grey areas in each figure indicate the relative aerial density of the insect (arb. unit), or the area of migrating insects 24 hours after their take-off.

Regarding the possible occurrence of overseas immigration during the survey periods in Japan, no *L*. *striatellus* were caught in the net trap at the Nagasaki Plant Protection Station, except the following small catches: one insect on May 30, 2012, May 25 and June 3, 2013, and 11 insects on June 4, 2013. In 2013, the daily total number of *L*. *striatellus* caught in the 10-m net trap at S1 also peaked on June 4. Examples of the migration simulation on the above four days in Nagasaki are shown in Fig. 9. The simulation predicted that no insects arrived in western Japan in the four cases, suggesting no overseas immigration. The simulation also indicated that no overseas migration of *L*. *striatellus* arriving in western Japan within 24 h should have been found during the entire survey periods in 2012 and 2013 (Table 3).

**Table 3:** Migration simulation results during the survey periods in Japan^a^.

Date	Occurrence of possible migration from Jiangsu Province to western Japan (Yes/No)	
	2012	2013
May 24	-	No
May 25	-	No
May 26	No	No
May 27	No	No
May 28	No	No
May 29	No	No
May 30	No	No
May 31	No	No
Jun 1	No	No
Jun 2	No	No
Jun 3	No	No
Jun 4	No	No
Jun 5	No	No
Jun 6	-	No
Jun 7	-	No
Jun 8	-	No

^a^ The Japanese survey periods in 2012 and 2013 were from May 26 to June 5, and from May 24 to June 8, respectively. Simulation method by Otuka et al. (2012) [19] was used. A number of simulated *L*. *striatellus* took off in Jiangsu Province at 1800 h CST on the previous day of each date in the table, and their flight duration was set to be 24 h [19]. See examples of the simulation in Fig. 9.

## Discussion

Did overseas migration occur during the survey periods at the Japanese sites in 2012 and 2013? The migration simulations in both years suggested there should have been no overseas migration from Jiangsu Province (Table 3). This prediction is reliable since the hitting ratio of the prediction (= the number of successful predictions/the total number of predictions) is over 90% [19]. Small numbers of the insects caught at the front of overseas immigration at Isahaya, located in the western part of Kyushu Island, were recorded in one day in 2012 and three days in 2013. The timing of emigration of the *L*. *striatellus* population in wheat fields around Isahaya was almost the same as that in the survey area in Saga, due to similar air temperature trends in the two areas (data not shown). Therefore, the insects caught at Isahaya should be attributed to the local population, not immigrants from overseas. Thus, no overseas immigration should have occurred at the Japanese sites during the survey periods in 2012 and 2013.

Since the patterns of insects caught in the net trap and the canopy trap in China corresponded to each other (Figs. 3 and 5), the catches in the net trap must have been local emigrants, not immigrants from outside of the local area. In a similar way, insects caught in traps in Japan (Fig. 7) must have consisted of local emigrants because the patterns in Figs. 3, 5 and 7a corresponded to each other and because no overseas immigration was suggested as described above. Therefore, take-off of the first generation of overwintering *L*. *striatellus* populations in mid-latitudes of East Asia occurred mainly between 1600 and 2000 h local time (Figs. 3, 5, 7 and 8). The peak of take-off time occurred at 1700 h (Fig. 5). Take-off in daytime between 0800 and 1600 h local time also occurred both in China and Japan. These characteristics were identical in western Japan and eastern China. There was no significant difference in the temporal pattern of take-off behavior between females and males (Fig. 7b, c). The difference seems noticeable in Fig. 5b because only a small number of insects were trapped. There was no catch peak at dawn, or when sampled at 0600 or 0700 h local time, in this study.

In addition, *L*. *striatellus* adults flying at about 260 m AGL were captured with the helicopter-towed net trap, although this part of the study could not be repeated due to weather and flight schedule. This finding might suggest that some portion of emigrating insects flew upwards after take-off similar to *N*. *lugens* [10, 31]. If the number of emigrants was large and winds at altitude were favorable for migration, some of the emigrants would be able to migrate long distances.

The flight periodicities of a variety of insects have been investigated and grouped into 42 types based on the timing of when insects are trapped in suction traps. For example, bimodal peaks appear at dusk and dawn, a single peak during the day and so on [25]. The flight periodicity of *L*. *striatellus* unveiled in this study belongs to type 13 in which gradually increasing numbers of insects are trapped during the day with a peak in the early evening before dusk. Since *Myzocallis castanicola* and *Myzus persicae* in the order Hemiptera and species in other taxa show the same periodicity, this pattern does not necessarily seem related to a certain taxon, but may depend on the insects’ ecological and physiological properties.

In a previous study of *L*. *striatellus*, the take-off times had bimodal peaks when yellow water-pan traps were used in rice paddy fields in early June, appearing both at 0800 h and 1800 h JST [22]. In that study, the pans were set at a height corresponding to the height of the rice plants, and insects walking or hopping into the pans as well as flying immigrants from outside of the field could be captured. Therefore, the results from the yellow water-pan traps do not necessarily indicate the insect’s flight activity; whereas, the canopy trap in our study could directly capture emigrants only when they took off. The morning flight activities around 0800 h local time as reported by Kisimoto (1968) [22] are evident in this study as well (Figs. 3, 5, and 6b), but of small magnitude. Peak numbers of insects trapped at 1800 h corresponds to the same evening peak found in this study. Thus, the patterns of flight activity of emigrants over wheat fields and immigrants in paddy fields were found to be slightly different. Peak numbers of insects caught at 1900–2100 h in the light trap in China [23, 24] also may be emigrants who took off in the evening as in this study and migrated for a few hours before arriving at the light trap. Small numbers of insects were caught in light traps in China at 0500 h [23, 24]. This result may be due to individuals that have already arrived around the light trap because dawn take-off during the wheat harvest season has been refuted in this study.

Tropical *N*. *lugens* shows a clear dusk and dawn take-off pattern in summer [20]. An illuminance of 100 lux is thought to be a trigger for the insect’s take-off [20]. In the case of *L*. *striatellus*, the major take-off peak around 1700 h JST was not related to an illuminance of 100 lux which occurred at later times past 1900 h JST. The take-off at dusk past 1900 h JST is evident (Fig. 6b and 6d), but its contribution to the total emigration number was small. The average surface air temperature at 2000 h JST at S1 from May 26 to June 4, 2012 was 22.6°C. Therefore, the surface air temperature at dusk may not be a limiting factor for the dusk take-off. At present, it is unclear why the majority of *L*. *striatellus* takes off earlier than *N*. *lugens*.

The bimodal take-off pattern of *N*. *lugens* changed as the air temperature dropped, and take-off in early October in western Japan occurred only at dusk or during the day [20, see a similar Chinese case in 31]. The number of *L*. *striatellus* trapped in this study was found to be low at dawn. The reason for this phenomenon is unknown, and a further study is needed to know how low air temperatures and other factors affect *L*. *striatellus*’s take-off at dawn in late May to early June, or other seasons as well.

After *L*. *striatellus* in the temperate zone of eastern China or Japan moves from a wheat host to rice plants, the population density of the subsequent one to two generations declines probably due to several factors, i.e., natural enemies, applied insecticides, and high temperatures in summer [32, 33]. In addition, rice plants in paddy fields are still healthy and suitable for food, and as a result, a mass emigration barely happens. Therefore, investigation of *L*. *striatellus* emigration in summer in the temperate zone seems to be rather difficult to conduct. On the other hand, in Taiwan which is located in the subtropical region, harvest of the first crop of rice, an environmental disturbance causing emigration, is conducted in late June to mid-July and *L*. *striatellus* is one of the major pests in rice fields. Thus, investigation of *L*. *striatellus* emigration timing in summer might be more feasible in Taiwan.

A diurnal take-off pattern was used to predict overseas migration of *L*. *striatellus* in East Asia. Take-off times at dusk and dawn were used in a recent migration simulation model [19]. Based on new knowledge generated by this study, take-off times in the evening and during the day should be implemented in the model. This modification may affect the prediction accuracy because changes in the take-off timing will affect the prediction of migration areas and arrival times under variable wind conditions. Currently, a new migration model is being evaluated.
